# Representation of Health Conditions on Facebook: Content Analysis and Evaluation of User Engagement

**DOI:** 10.2196/jmir.3275

**Published:** 2014-08-04

**Authors:** Timothy M Hale, Akhilesh S Pathipati, Shiyi Zan, Kamal Jethwani

**Affiliations:** ^1^Center for Connected HealthPartners HealthCare, IncHarvard Medical SchoolBoston, MAUnited States; ^2^Stanford University School of MedicineStanford, CAUnited States

**Keywords:** Internet, Facebook, social media, social networking sites, social support, health communication, information seeking behavior

## Abstract

**Background:**

A sizable majority of adult Internet users report looking for health information online. Social networking sites (SNS) like Facebook represent a common place to seek information, but very little is known about the representation and use of health content on SNS.

**Objective:**

Our goal in this study was to understand the role of SNS in health information seeking. More specifically, we aimed to describe how health conditions are represented on Facebook Pages and how users interact with these different conditions.

**Methods:**

We used Google Insights to identify the 20 most searched for health conditions on Google and then searched each of the resulting terms on Facebook. We compiled a list of the first 50 Facebook “Pages” results for each health condition. After filtering results to identify pages relevant to our research, we categorized pages into one of seven categories based on the page’s primary purpose. We then measured user engagement by evaluating the number of “Likes” for different conditions and types of pages.

**Results:**

The search returned 50 pages for 18 of the health conditions, but only 48 pages were found for “anemia” and 5 pages were found for “flu symptoms”, yielding a total of 953 pages. A large number of pages (29.4%, 280/953) were irrelevant to the health condition searched. Of the 673 relevant pages, 151 were not in English or originated outside the United States, leaving 522 pages to be coded for content. The most common type of page was marketing/promotion (32.2%, 168/522) followed by information/awareness (20.7%, 108/522), Wikipedia-type pages (15.5%, 81/522), patient support (9.4%, 49/522), and general support (3.6%, 19/522). Health conditions varied greatly by the primary page type. All health conditions had some marketing/promotion pages and this made up 76% (29/38) of pages on acquired immunodeficiency syndrome (AIDS). The largest percentage of general support pages were cancer (19%, 6/32) and stomach (16%, 4/25). For patient support, stroke (67%, 4/6), lupus (33%, 10/30), breast cancer (19%, 6/31), arthritis (16%, 6/36), and diabetes (16%, 6/37) ranked the highest. Six health conditions were not represented by any type of support pages (ie, human papillomavirus, diarrhea, flu symptoms, pneumonia, spine, human immunodeficiency virus). Marketing/promotion pages accounted for 46.73% (10,371,169/22,191,633) of all Likes, followed by support pages (40.66%, 9,023,234/22,191,633). Cancer and breast cancer accounted for 86.90% (19,284,066/22,191,633) of all page Likes.

**Conclusions:**

This research represents the first attempts to comprehensively describe publicly available health content and user engagement with health conditions on Facebook pages. Public health interventions using Facebook will need to be designed to ensure relevant information is easy to find and with an understanding that stigma associated with some health conditions may limit the users’ engagement with Facebook pages. This line of research merits further investigation as Facebook and other SNS continue to evolve over the coming years.

## Introduction

The Internet has radically changed how most people find and share information about health and medical conditions. The practice of looking for health information online has become increasingly popular, with 59% of US adults (72% of adult Internet users) reporting that they have done so in the past year [1]. Nearly half of these individuals reported that the information they found online led them to believe they needed to seek health attention [1]. Even for serious health conditions such as cancer, people are more likely to turn to the Internet first for health information despite a greater trust in their doctor as a source of information [2]. There are many reasons for the rise of the Internet as a source of health information, including: 24/7 availability, the greater anonymity it offers for those with sensitive health care needs, and the opportunity to locate and connect with other people with similar health conditions [3].

Social media is a relatively new health communication channel that enables people to communicate and interact with a larger number of people, find and share information about their health and medical conditions, and receive health messages [4]. Social networking sites (SNS) are one of the most popular and widely used forms of social media with 72% of online US adults using SNS, as of May 2013 [5]. Facebook is the most widely used SNS [6], with 93% of online US adult users reporting having a Facebook account [5] and with 727 million daily users worldwide [7]. Facebook began as an online social network for college students and remains popular with young adults—86% of Internet users aged 18-29 years use Facebook [6]. There has been a significant upward trend in its adoption by older adults in recent years and now 73% of Internet users aged 30 to 49 years and 57% of those aged 50 to 64 years report using Facebook [6]. This is especially significant considering that individuals become more likely to develop chronic health conditions as they age.

Despite the rapid and widespread adoption of Facebook among Internet users, little is known about the broader representation of health conditions on Facebook. The existing literature has largely focused on a small number of specific health conditions that have taken one of two approaches, either (1) an aggregated content analysis of posts, or (2) a more detailed analysis of differences in the primary purpose of groups, the number of members, and the content of posts.

Studies examining the content of posts find marked differences by health condition. Greene et al [8] examined diabetes groups and found that two-thirds of wall posts and discussion topics were characterized by sharing of information on diabetes management strategies, followed by posts related to emotional support and promotional themes. In contrast to the findings for diabetes groups, Ahmed et al [9] found that among Facebook groups devoted to concussions, nearly two-thirds of posts were to relate personal experiences of a concussion and posts were only rarely used to seek information (8%) or offer advice (2%). Gajaria et al [10] examined posts by youth to attention deficit hyperactivity disorder (ADHD) Facebook groups. They found the largest percentage of posts (42%) were about defining ADHD and creating a sense of group identity, and to seek and share advice regarding medications and symptom management (35%).

Bender et al and Thoren et al examined types of groups, number of members, and the content of posts. Bender et al [11] examined the content of breast cancer groups and found that most groups were created for fundraising or awareness purposes, rather than supportive care. They also found that the awareness groups had the most members, while the support groups generated the greatest number of posts. Thoren et al [12] examined the content of Facebook groups focusing on premature infants. Similar to findings from breast cancer groups, they found that most premature infant groups were devoted to fundraising or awareness purposes and that these groups had the most members. However, despite the emphasis on fundraising and awareness groups, 53% of all posts were for “interpersonal support” and 31% for “information sharing”.

To our knowledge, only two studies have been conducted to characterize the representation of a broader range of health conditions on Facebook [13,14]. The most comprehensive study was conducted by Farmer et al [14], who constructed a list of search terms based on the 11 most prevalent non-communicable diseases identified by the World Health Organization. Using both health and lay terms, they searched all Facebook groups between December 2007 and January 2009. They found that respiratory groups, diabetes, cardiovascular disease, and digestive disease made up the largest number of groups, while groups related to malignant neoplasms had the most members. Patient groups comprised of disease sufferers were the most common (47%), followed by support (28%), and fundraising (19%) groups. De la Torre-Díez et al [13] examined how three diseases with the greatest public burden (ie, breast cancer, colorectal cancer, and diabetes) are represented on Facebook and Twitter. Conducting a search in 2011, they found that “prevention” groups that seek to raise awareness and/or money of a disease was the most popular categorization for all three diseases (18%), followed by support groups (17.9%), and research investigations (14.3%).

Taken together, these studies have begun to demonstrate how people use Facebook to find and share health information. However, these findings fail to reflect the representation of health conditions on Facebook, due to the focus on specific health conditions or the limited inclusion criteria for disease groups (ie, non-communicable diseases, diseases with greatest public burden). Therefore, we still lack a comprehensive review of how health conditions are represented on Facebook.

In this paper, we aimed to (1) describe the results of a search for 20 common health conditions on Facebook “Pages”, (2) identify the purpose and content of these pages, and (3) evaluate user engagement with these pages.

Results may offer important insights for future public health initiatives. For example, a better understanding of which conditions are prominent on Facebook provides perspective on the accessibility of information on different diseases. Second, variation in accessibility may have further implications for class-specific engagement with health conditions on Facebook. Finally, data on user engagement may provide a means for health professionals to more effectively disseminate information on Facebook.

## Methods

### Facebook Pages

Unlike previous studies, we chose to focus our search on Facebook “Pages” rather than “Groups”. In the evolution of Facebook, groups initially served as a primary forum for communication and, as such, many of the previous studies cited above conducted their research within this realm. However, the functionality of groups began to shift following the introduction of Facebook Pages in 2007, initially created as a way to allow public profile owners (individuals, organizations, services, etc) to advertise to Facebook users more easily. These “Fan Pages” behaved much like a user’s profile and allowed owners to send updates to those who subscribed to their page and access insights and analytics of their fan base. Until April 19, 2010, users had the option to become a “Fan” of a page; this subsequently changed so that users could “Like” a page. These “Like Pages” allow for an unlimited number of “Likers” and have additional functionalities including the ability to add tabs for email collection and specialized content. In addition, “Community Pages” were also introduced around this time, allowing for the integration of content into Facebook directly from Wikipedia pages.

Pages and groups differ by their function: pages can be thought to resemble a promotional blog, whereas groups are more analogous to a moderated message board. The key benefits of a page over a group is that pages (1) are able to get internal promotion through the page feed of fans after they like a page, (2) have more options for customization, (3) have greater search engine visibility, (4) allow the creator or administrator of the page to remain anonymous, and (5) give the user more power to control the content they receive from the page.

In more recent years, pages have exploded in popularity as a means for publicly accessible interaction, to the point that some social media commentators have even described groups as “obsolete” or as a “Facebook fossil” [15]. Along with this shift, more and more groups have become “closed” (visible on Facebook but content is visible to members only) or “secret” (completely invisible to all on Facebook except for invited members), making information contained within groups no longer easily accessible to a casually browsing Facebook user. Therefore, we decided to focus our study on pages, as we believe that it would be able to provide a more complete picture of a health condition’s representation on Facebook.

### Search Criteria and Strategy

On July 24, 2012, we identified the 20 most searched for health conditions on Google using Google Insights (see Table 1). These 20 conditions provided the basis for our subsequent searches on Facebook. On the same day, we conducted searches for these 20 health conditions on Facebook using Facebook Search. For our searches, we specifically focused on Facebook pages, excluding search results for people, groups, and other categories. We recorded the top 50 pages results for each health condition, as well as the URL and the number of Likes each page had received from Facebook members.

The Facebook search algorithm is user-centric and search results will vary for different people based on their past Facebook use, profile information, and network of friends [16]. To minimize this effect, we created a new Facebook account using minimal biographical information, specifying only name, gender, and age: Jonathan Davis, male, 45 years old. By creating a generic profile, we hoped to retrieve search results that would be more representative of health conditions on Facebook and that are not tailored to individual factors, social context, or geographical location of the person searching. We ensured that our new profile had no friends and no preexisting Likes. We also deleted and disabled cookies and location services prior to conducting our searches.

**Figure 1:**
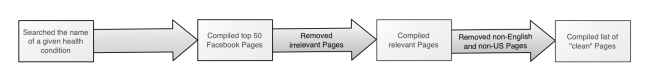
Search result workflow.

Once we had compiled lists of 50 pages for each search term, we filtered our search results to limit our analysis to those pages that were relevant to the health condition (see Figure 1). For instance, when we conducted our search for human immunodeficiency virus (HIV), many of the top 50 pages were fan pages for a band named “The Hive”. Similarly, many results for diarrhea were for a band named “Raging Diarrhea”, or other topics irrelevant to the health condition. We then further restricted our analysis to pages that were in English, and that were based in the United States. If a country was not specified but the page was in English, we assumed that it was based in the United States. After filtering the pages with our criteria, we generated a list of “clean” pages for each condition, which listed the name of each page and the number of Likes it had received.

**Table 1:** Google Insights results: top 20 health condition search terms used in the United States between September 2007-June 2012.^a^

Google Insights	Health condition
1	cancer
2	diabetes
3	stomach
4	herpes
5	back pain
6	human immunodeficiency virus (HIV)
7	blood pressure
8	thyroid
9	breast cancer
10	arthritis
11	acquired immunodeficiency syndrome (AIDS)
12	lupus
13	diarrhea
14	pneumonia
15	spine
16	flu symptoms
17	human papilloma virus (HPV)
18	asthma
19	anemia
20	stroke

^a^Search conducted on July 24, 2012.

### Coding Page Content and Descriptive Analysis

To determine categories for classifying pages, we started with a literature review to identify previous categorizations of groups on Facebook. As noted in the introduction, Facebook pages and groups serve somewhat different roles, but previous research provided a starting point for identifying their purpose. For example, Greene et al [8] used five categories: advertisements, providing information, requesting information, support, and irrelevant; De la Torre-Díez et al [13] identified five categories: fund collecting, awareness, support, prevention, and disease-fighting; Bender et al [11] used four categories: fundraising, awareness, promote-a-site, and support; and Farmer et al [14] used four categories: patient groups, support groups, fundraising/charity groups, and other. Based on this review, we initially chose to cluster pages into five categories: patient support, general support, information/awareness, marketing/promotion, and other.

**Figure 2:**
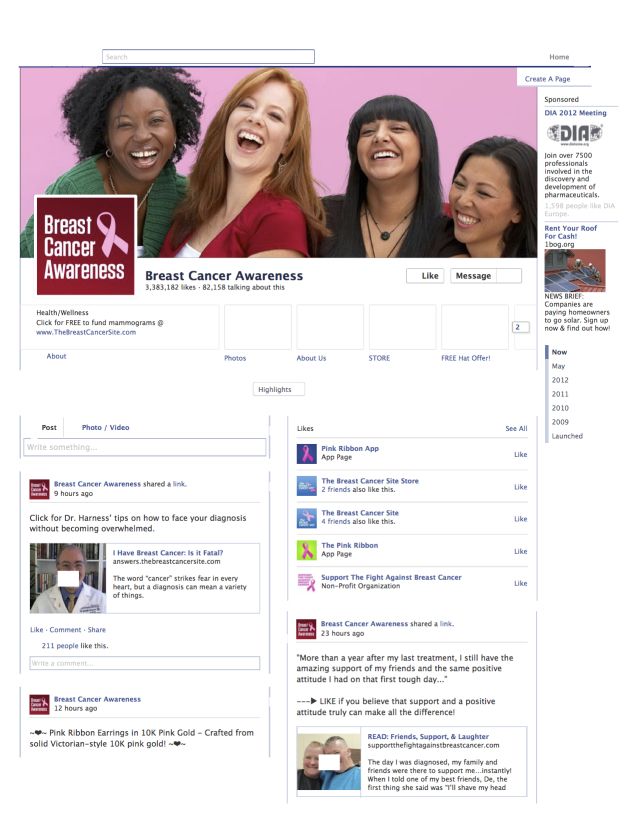
Screenshot of a Facebook page analyzed in this study.

Once we compiled our list of pages, two co-authors (ASP and SZ) evaluated the 20 most recent posts on each page and categorized page content into one of five types. An example Facebook page is presented in Figure 2. The two coders conducted an initial categorization of approximately 90 pages in order to determine interrater reliability (IRR). Although the IRR was acceptable (Cohen’s kappa=.74), there was disagreement on how to code pages that lacked content, or were Wikipedia-type informational pages with no user content. As a result, we added categories for Wikipedia and blank pages, giving us seven categories (see Table 2). Another 90 pages were coded using the new classification scheme and the IRR improved (Cohen’s kappa=.83). The remaining pages were then divided between the two coders.

Once all pages were coded, data was aggregated for each condition. We first aggregated data on the number of pages by health condition and type of content. We then compiled data on the number of page Likes by condition and calculated the total number of Likes by page content. We used the number of Likes as a proxy for member interest or engagement with a health condition on Facebook.

**Table 2:** Page categorization and example posts.

Categorization	Description	Example page and post
1. Patient Support	Characterized by emotional and informational support for patients of the condition. Often included motivational messages, links, and posts by affected individuals.	Kick Cancer “No matter how scary things may seem or how bad they may look, keep going and never give up! #kickcancer”
2. General Support	Characterized by emotional and informational support for caregivers, family, friends, and some patients themselves. Often included motivational messages and posts by supporters of affected individuals.	Mesothelioma Cancer Alliance “I hope they find a way to diagnose this disease sooner so more people can be cured and not have to go thru the devastation so many of us have had to go thru. Rest in peace daddy.”
3. Marketing/Promotion	Characterized by promotion of specific products, events, or institutions. Included self-promotion of the page or events and organization run by the page managers.	Cancer Sucks “New Cancer Sucks Purple Heart design tee’s available in both men’s and women’s styles! Get yours now!”
4. Information/Awareness	Characterized by a focus on raising awareness of a condition or facilitating information exchange. Included many links to information, treatment recommendations, and research pages.	Breast Cancer Awareness “medicalxpress.com: Accelerated radiation treatment effective for noninvasive breast cancer”
5. Wikipedia	Some pages simply provided information from Wikipedia on the condition.	Cancer “From Wikipedia, the free encyclopedia”
6. Blank	Blank pages were pages that addressed the condition, but did not have any posts.	Diarrhea None
7. Other	Any pages that did not fit the above categories were classified as “other.” For example, we classified personal blogs by people who were affected by a condition as “other”.	Mrs Lupus “Here is one of my most popular blog posts, it is not very informative but it is written with raw emotion that all us lupies can understand.”

## Results

### Facebook Page Search

Our first aim was to describe the results of a search for 20 common health conditions on Facebook. We used the Facebook search function to list the first 50 pages found for each of the 20 health conditions identified using Google Insights (see Table 3). The search returned 50 pages for 18 of the health conditions, but only 48 pages were found for “anemia” and five pages were found for “flu symptoms”. Thus, the list or sample of pages returned was 953 pages.

The Facebook search turned up a number of irrelevant pages that were not about health conditions. Of the 953 pages returned in the search, 280 pages were not about the health condition used in the search term. The number of relevant pages also varied considerably by health condition. Conditions with the greatest number of relevant pages were breast cancer and diabetes (n=50), followed by cancer, thyroid, and arthritis (n=49). Conditions with the lowest number of relevant pages were stroke (n=10), HIV (n=10), spine (n=18), human papillomavirus (HPV; n=23), and diarrhea (n=23). The search for flu symptoms yielded only five pages, but 100% of the pages were relevant.

A second criteria was that pages be in English and have a user base located in the United States. This further reduced the number of relevant pages by 151 to 522 pages. Five conditions (stroke, HPV, asthma, breast cancer, and cancer) had one-third or more of relevant pages in a language other than English or located outside North America. The median number of relevant pages for each health condition was 29 and ranged from 5 to 43 pages (results not shown in Table 3).

**Table 3:** Relevant Facebook pages.

Google Insights ranking	Health condition	Pages sampled	Relevant, n (%)	Eliminated non-US, n (%)^a^	Clean pages^b^, n (%)^c^
1	cancer	50	49 (98.0)	17 (34.7)	32 (64.0)
2	diabetes	50	50 (100.0)	13 (26.0)	37 (74.0)
3	stomach	50	26 (52.0)	1 (3.9)	25 (50.0)
4	herpes	50	33 (66.0)	5 (15.2)	28 (56.0)
5	back pain	50	44 (88.0)	10 (22.7)	34 (68.0)
6	human immunodeficiency virus (HIV)	50	10 (20.0)	2 (20.0)	8 (16.0)
7	blood pressure	50	43 (86.0)	6 (14.0)	37 (74.0)
8	thyroid	50	49 (98.0)	6 (12.2)	43 (86.0)
9	breast cancer	50	50 (100.0)	19 (38.0)	31 (62.0)
10	arthritis	50	49 (98.0)	13 (26.5)	36 (72.0)
11	acquired immunodeficiency syndrome (AIDS)	50	43 (86.0)	5 (11.6)	38 (76.0)
12	lupus	50	37 (74.0)	7 (18.9)	30 (60.0)
13	diarrhea	50	23 (46.0)	4 (17.4)	19 (38.0)
14	pneumonia	50	39 (78.0)	3 (7.7)	36 (72.0)
15	spine	50	18 (36.0)	4 (22.2)	14 (28.0)
16	flu symptoms	5	5 (100.0)	0 (0.0)	5 (100.0)
17	human papillomavirus (HPV)	50	23 (46.0)	9 (39.1)	14 (28.0)
18	asthma	50	41 (82.0)	16 (39.0)	25 (50.0)
19	anemia	48	31 (64.6)	7 (22.6)	24 (50.0)
20	stroke	50	10 (20.0)	4 (40.0)	6 (12.0)
Totals		953	673 (70.6)	151 (22.4)	522 (54.8)

^a^Percent of health condition relevant pages.

^b^Mean number of pages=26; median number of pages=29; interquartile range=18.25.

^c^Percent of pages sampled.

### Health Conditions on Facebook Pages

Our second aim was to identify the content of the 522 pages identified as relevant to health conditions. The most frequent page type was marketing/promotion, which accounted for 168/522 or 32.2% of pages. The next most frequent page types were information and awareness of a health condition (20.7%, 108/522) followed by Wikipedia-type pages (15.5%, 81/522), patient support (9.4%, 49/522), and general support (3.6%, 19/522). A total of 64 pages were coded as “other” type. Finally, 33 pages that did not contain enough information were coded as blank.

Next, we examined pages by health condition and content (see Tables 4 and 5). All health conditions had some pages devoted to marketing/promotion, ranging from 76% (29/38) of acquired immunodeficiency syndrome (AIDS) pages to 5% (1/19) of diarrhea pages. Six conditions (ie, AIDS, arthritis, spine, breast cancer, asthma, and cancer) had more than 40% of the pages devoted primarily to marketing/promotion. Wikipedia pages comprised a large percentage of acute conditions (anemia, 58%, 14/24; pneumonia, 58%, 21/36; flu symptoms, 40%, 2/5; and diarrhea, 26%, 5/19) but formed a much smaller proportion for breast cancer (3%, 1/31) and cancer (3%, 1/32) pages. For HIV, spine, and diabetes pages, 40% or more were information/awareness-type pages. In contrast to the high percentage of Wikipedia pages devoted to pneumonia, diarrhea, and flu symptoms, these conditions made up a small percentage of information/awareness pages. Cancer and breast cancer made up 25% (8/32) and 23% (7/31) of information/awareness pages, respectively. Conditions with the largest percentage of support pages were cancer (19%, 6/32) and stomach (16%, 4/25) for general support, and stroke (67%, 4/6), lupus (33%, 10/30), breast cancer (19%, 6/31), arthritis (16%, 6/36), and diabetes (16%, 6/37) for patient support. A large number of health conditions were not represented by any type of support pages (ie, HPV, diarrhea, flu symptoms, pneumonia, spine, HIV). Over 30% of blood pressure, diarrhea, and herpes pages could not be classified as one of the other types and were categorized under other. Over 25% of HPV and diarrhea pages did not contain sufficient information to determine the purpose of the pages and were categorized as blank.

**Table 4:** Facebook pages by health condition and content—General Support, Patient Support, and Information/Awareness.

Google Insights ranking	Health condition	Clean pages, n	General support, n (%)	Patient support, n (%)	Information, n (%)
1	cancer	32	6 (18.8)	3 (9.4)	8 (25.0)
2	diabetes	37	1 (2.7)	6 (16.2)	15 (40.5)
3	stomach	25	4 (16.0)	1 (4.0)	7 (28.0)
4	herpes	28	1 (3.6)	0 (0.0)	4 (14.3)
5	back pain	34	0 (0.0)	3 (8.8)	11 (32.4)
6	human immunodeficiency virus (HIV)	8	0 (0.0)	0 (0.0)	4 (50.0)
7	blood pressure	37	0 (0.0)	1 (2.7)	10 (27.0)
8	thyroid	43	0 (0.0)	5 (11.6)	11 (25.6)
9	breast cancer	31	1 (3.2)	6 (19.4)	7 (22.6)
10	arthritis	36	1 (2.8)	6 (16.7)	3 (8.3)
11	acquired immunodeficiency syndrome (AIDS)	38	3 (7.9)	0 (0.0)	3 (7.9)
12	lupus	30	1 (3.3)	10 (33.3)	5 (16.7)
13	diarrhea	19	0 (0.0)	0 (0.0)	1 (5.3)
14	pneumonia	36	0 (0.0)	0 (0.0)	2 (5.6)
15	spine	14	0 (0.0)	0 (0.0)	6 (42.9)
16	flu symptoms	5	0 (0.0)	0 (0.0)	0 (0.0)
17	human papillomavirus (HPV)	14	0 (0.0)	0 (0.0)	2 (14.3)
18	asthma	25	1 (4.0)	2 (8.0)	7 (28.0)
19	anemia	24	0 (0.0)	2 (8.3)	2 (8.3)
20	stroke	6	0 (0.0)	4 (66.7)	0 (0.0)
Total		522	19 (3.6)	49 (9.4)	108 (20.7)

**Table 5:** Facebook pages by health condition and content—Wikipedia, Marketing, Other, and Blank.

Google Insights ranking	Health condition	Clean pages, n	Wikipedia, n (%)	Marketing, n (%)	Other, n (%)	Blank, n (%)
1	cancer	32	1 (3.1)	13 (40.6)	0 (0.0)	1 (3.1)
2	diabetes	37	3 (8.1)	11 (29.7)	1 (2.7)	0 (0.0)
3	stomach	25	2 (8.0)	3 (12.0)	5 (20.0)	3 (12.0)
4	herpes	28	5 (17.9)	5 (17.9)	9 (32.1)	4 (14.3)
5	back pain	34	2 (5.9)	10 (29.4)	6 (17.7)	2 (5.9)
6	human immunodeficiency virus (HIV)	8	2 (25.0)	2 (25.0)	0 (0.0)	0 (0.0)
7	blood pressure	37	5 (13.5)	4 (10.8)	14 (37.8)	3 (8.1)
8	thyroid	43	7 (16.3)	9 (20.9)	8 (18.6)	3 (7.0)
9	breast cancer	31	1 (3.2)	16 (51.6)	0 (0.0)	0 (0.0)
10	arthritis	36	3 (8.3)	22 (61.1)	1 (2.8)	0 (0.0)
11	acquired immunodeficiency syndrome (AIDS)	38	2 (5.3)	29 (76.3)	1 (2.6)	0 (0.0)
12	lupus	30	0 (0.0)	11 (36.7)	3 (10.0)	0 (0.0)
13	diarrhea	19	5 (26.3)	1 (5.3)	7 (36.8)	5 (26.3)
14	pneumonia	36	21 (58.3)	2 (5.6)	4 (11.1)	7 (19.4)
15	spine	14	0 (0.0)	8 (57.1)	0 (0.0)	0 (0.0)
16	flu symptoms	5	2 (40.0)	1 (20.0)	1 (20.0)	1 (20.0)
17	human papillomavirus (HPV)	14	4 (28.6)	3 (21.4)	1 (7.1)	4 (28.6)
18	asthma	25	1 (4.0)	12 (48.0)	2 (8.0)	0 (0.0)
19	anemia	24	14 (58.3)	5 (20.9)	1 (4.2)	0 (0.0)
20	stroke	6	1 (16.7)	1 (16.7)	0 (0.0)	0 (0.0)
Total		522	81 (15.5)	168 (32.2)	64 (12.3)	33 (6.3)

### Likes on Facebook Pages

Our third aim was to examine the level of user engagement for each of the 20 health conditions represented on Facebook pages. For each of the conditions, we aggregated the number of Likes for the pages of each condition, as well as the average, median, minimum, and maximum (see Table 6). For the 20 health conditions we searched for on Facebook pages, there were 22,191,633 Likes. The mean number of Likes across all health condition pages was 1,110,240 and ranged from 0 to 3,537,360. Cancer and breast cancer together account for 86.90% (19,284,066/22,191,633) of total Likes. AIDS and diabetes each account for about 4.5% of total Likes, followed by HIV and lupus with about 1.1% each. The remaining 14 health conditions represent less than 2% of the total Likes.

Likes were most often given to marketing/promotion pages, which accounted for 46.73% (10,371,169/22,191,633) of all Likes. Support pages accounted for 40.66% (9,023,234/22,191,633) of total Likes with general support accounting for 35.89% (7,964,328/22,191,633) and patient support for 4.77% (1,058,906/22,191,633). This is in contrast to the findings for the number of pages, in which patient support and general support accounted for relatively small percentages of the total pages (9.4%, 49/522 and 3.6%, 19/522, respectively) compared to information and Wikipedia pages (20.7%, 108/522 and 15.5%, 81/522, respectively).

Finally, we examined how the number of Likes by health condition and type of page content (see Tables 7 and 8). Twelve health conditions were primarily represented by Likes on marketing/promotion pages, with the percentage of pages coded as marketing/promotion exceeding the percentage for any other type (herpes, HIV, HPV, AIDS, flu symptoms, anemia, cancer, lupus, breast cancer, spine, blood pressure, and back pain). A total of 80% or more of herpes, HIV, and HPV Likes were on pages coded as marketing/promotion. Seven conditions were primarily represented by Likes on information/awareness or Wikipedia pages. Pneumonia, diabetes, arthritis, thyroid, and stomach were primarily represented by Likes on information/awareness pages. Only 9-10% of cancer and breast cancer Likes were on information/awareness pages. Cancer and breast cancer Likes were nearly evenly divided between marketing/promotion and combined support pages. Cancer and breast cancer pages accounted for most of the general support Likes (94.59%, 7,533,563/7,964,328) and, although a modest percentage of breast cancer and cancer pages were categorized as patient support (19%, 6/31 and 9%, 3/32 respectively), the large number of Likes for these two health conditions comprised 82.78% (876,589/1,058,906) of the total number of patient support Likes. Wikipedia pages made up the largest percentage of diarrhea and asthma Likes. The largest percentage of Likes on thyroid pages were on those classified as patient support.

**Table 6:** Facebook “Likes” by health condition.

Google Insights ranking	Health condition	Total Likes, n (%)	Average	Minimum	Maximum	Median
1	cancer	10,228,611 (46.09)	319,644	956	3,537,341	29,776
2	diabetes	986,868 (4.45)	26,672	618	473,585	4529
3	stomach	28,620 (0.13)	1145	202	6044	572
4	herpes	46,778 (0.21)	1671	3	36,730	104
5	back pain	15,165 (0.07)	446	1	2299	282
6	human immunodeficiency virus (HIV)	249,468 (1.12)	31,184	246	207,098	4215
7	blood pressure	9711 (0.04)	262	0	1629	27
8	thyroid	75,539 (0.34)	1757	2	14,249	815
9	breast cancer	9,055,455 (40.81)	292,111	1,005	3,537,360	19,054
10	arthritis	106,565 (0.48)	2960	200	34,578	708
11	acquired immunodeficiency syndrome (AIDS)	1,014,419 (4.57)	26,695	651	551,888	3487
12	lupus	241,286 (1.09)	8043	231	76,297	1712
13	diarrhea	6215 (0.03)	327	2	2329	44
14	pneumonia	6952 (0.03)	193	0	4405	3
15	spine	38,213 (0.17)	2569	197	13,196	588
16	flu symptoms	43 (0.00)	9	0	27	2
17	human papillomavirus (HPV)	5371 (0.02)	384	0	3594	57
18	asthma	24,890 (0.11)	996	192	8279	390
19	anemia	11,887 (0.05)	495	7	2845	190
20	stroke	39,577 (0.18)	6596	2129	18,670	3915
Total		22,191,633 (100.00)				

**Table 7:** Facebook “Likes” by health condition and content—General Support, Patient Support, and Information/Awareness.

Google Insights ranking	Health condition	Total Likes	General support, n (%)	Patient support, n (%)	Information, n (%)
1	cancer	10,228,611	3,996,203 (39.07)	372,880 (3.65)	905,348 (8.85)
2	diabetes	986,868	207,285 (21.00)	71,482 (7.24)	559,877 (56.73)
3	stomach	28,620	6466 (22.59)	2022 (7.06)	8439 (29.49)
4	herpes	46,778	32 (0.07)	0 (0.00)	3772 (8.06)
5	back pain	15,165	0 (0.00)	1567 (10.33)	3662 (24.15)
6	human immunodeficiency virus (HIV)	249,468	0 (0.00)	0 (0.00)	28,815 (11.55)
7	blood pressure	9711	0 (0.00)	992 (10.22)	3868 (39.83)
8	thyroid	75,539	0 (0.00)	4700 (6.22)	22,761 (30.13)
9	breast cancer	9,055,455	3,537,360 (39.06)	503,709 (5.56)	888,712 (9.81)
10	arthritis	106,565	2124 (1.99)	30,204 (28.34)	42,929 (40.28)
11	acquired immunodeficiency syndrome (AIDS)	1,014,419	214,308 (21.13)	0 (0.00)	19,297 (1.90)
12	lupus	241,286	231 (0.10)	37,287 (15.45)	79,861 (33.10)
13	diarrhea	6215	0 (0.00)	0 (0.00)	1433 (23.06)
14	pneumonia	6952	0 (0.00)	0 (0.00)	4455 (64.08)
15	spine	38,213	0 (0.00)	0 (0.00)	22,885 (59.89)
16	flu symptoms	43	0 (0.00)	0 (0.00)	0 (0.00)
17	human papillomavirus (HPV)	5371	0 (0.00)	0 (0.00)	121 (2.25)
18	asthma	24,890	319 (1.28)	1460 (5.87)	7927 (31.85)
19	anemia	11,887	0 (0.00)	101 (0.85)	1240 (10.43)
20	stroke	39,577	0 (0.00)	32,502 (82.12)	0 (0.00)
Total		22,191,633	7,964,328 (35.87)	1,058,906 (4.77)	2,605,402 (11.73)

**Table 8:** Facebook “Likes” by health condition and content—Wikipedia, Marketing, Other, and Blank.

Google Insights ranking	Health condition	Total Likes	Wikipedia, n (%)	Marketing, n (%)	Other, n (%)	Blank, n (%)
1	cancer	10,228,611	20,428 (0.20)	4,932,796 (48.23)	0 (0.00)	956 (0.01)
2	diabetes	986,868	36,875 (3.74)	110,731 (11.22)	618 (0.06)	0 (0.00)
3	stomach	28,620	2232 (7.80)	2868 (10.02)	5387 (18.82)	1206 (4.21)
4	herpes	46,778	1412 (3.02)	40,495 (86.57)	810 (1.73)	257 (0.55)
5	back pain	15,165	2726 (17.98)	4701 (31.00)	364 (2.40)	2145 (14.14)
6	human immunodeficiency virus (HIV)	249,468	8966 (3.59)	211,687 (84.86)	0 (0.00)	0 (0.00)
7	blood pressure	9711	1320 (13.59)	3023 (31.13)	504 (5.19)	4 (0.04)
8	thyroid	75,539	9313 (12.33)	19,883 (26.32)	18,850 (24.95)	32 (0.04)
9	breast cancer	9,055,455	1757 (0.02)	4,123,917 (45.54)	0 (0.00)	0 (0.00)
10	arthritis	106,565	9021 (8.47)	17,782 (16.69)	4505 (4.23)	0 (0.00)
11	acquired immunodeficiency syndrome (AIDS)	1,014,419	25,327 (2.50)	751,491 (74.08)	3996 (0.39)	0 (0.00)
12	lupus	241,286	0 (0.00)	114,832 (47.59)	9075 (3.76)	0 (0.00)
13	diarrhea	6215	2436 (39.20)	321 (5.16)	1956 (31.47)	69 (1.11)
14	pneumonia	6952	1705 (24.53)	605 (8.70)	167 (2.40)	20 (0.29)
15	spine	38,213	0 (0.00)	15,328 (40.11)	0 (0.00)	0 (0.00)
16	flu symptoms	43	13 (30.23)	27 (62.79)	1 (2.33)	2 (4.65)
17	human papillomavirus (HPV)	5371	790 (14.71)	4329 (80.60)	7 (0.13)	124 (2.31)
18	asthma	24,890	8279 (33.26)	5134 (20.63)	1771 (7.12)	0 (0.00)
19	anemia	11,887	4103 (34.52)	6273 (52.77)	170 (1.43)	0 (0.00)
20	stroke	39,577	2129 (5.38)	4946 (12.50)	0 (0.00)	0 (0.00)
Total		22,191,633	138,832 (0.63)	10,371,169 (46.71)	48,181 (0.22)	4815 (0.02)

## Discussion

### Principal Findings

We used Google Insights to identify the 20 most searched for health conditions and then searched for these terms on Facebook. We found that a large number of pages were not about the health condition searched, but a similarly named topic. The most common type of page content was marketing/promotion, followed by information/awareness. Only a small number of pages were devoted to social support and six conditions were not represented by any support pages (ie, HPV, diarrhea, flu symptoms, pneumonia, spine, HIV). We also found that engagement measured by Likes was greater for general support and marketing/promotion than for patient support and information/awareness pages.

### Relevant Pages

A Facebook search for health conditions returned a large number of page results that were not relevant to the health condition searched (29.4%, 280/953). Additionally, the percentage of relevant pages varies considerably by health condition. While 98% or more of pages listed for six conditions were relevant (flu symptoms, diabetes, breast cancer, cancer, thyroid, arthritis), less than 50% of pages were relevant for five conditions (HIV, stroke, spine, HPV, diarrhea; see Tables 4 and 5).

The variation in the number of relevant pages may be due to the breadth of health conditions that we searched for and/or the method used to identify the Facebook groups and pages. Previous research examining Facebook groups for specific health conditions found that most content was relevant. A total of 97% of the posts were relevant on the 25 largest Facebook groups, focusing on premature infants [12] and on the largest Facebook diabetes groups [8]. Ahmed and colleagues [9] found that 89% of posts on 17 Facebook groups related to concussions were relevant. In contrast, using a search method similar to the one we used, Sajadi and Goldman [17] examined the usefulness of the first 30 listed results for the search term “incontinence” on Facebook, Twitter, and YouTube. They found that nearly half of the search results on Facebook led to pages with no useful information. This problem may be overrepresented in our study due to our search methodology of using a “clean” Facebook profile. With more information about a user, Facebook is likely to show pages that are more relevant to the user, which may also be more relevant to the condition searched.

The difficulty in finding Facebook pages with relevant health information may pose a significant barrier for people with inadequate digital skills. A growing body of literature finds that people with better Internet skills are more likely to go online to search for information, including health information [18,19], and make more varied and effective use of online information resources [20]. Additionally, Internet and information seeking skills vary by socioeconomic status and prior Internet access and use [19,20]. These digital inequalities may limit the utility of Facebook as a health communication channel for people from socially disadvantaged groups and may in fact contribute to increasing knowledge gaps [21-24] and health disparities. Public health interventions that use Facebook as a health communication channel will need to be designed to ensure that information is easy to find for all members of the target population.

### Page Content and Social Support

One benefit of using social media for health communication, identified by Moorhead et al [4] in a systematic review of 98 research articles, is the ability for people to draw social support from a large network of friends, relatives, and other users. We found, however, that only 13.0% (68/522) of pages were devoted to social support and that the largest percentage of pages were marketing/promotion (32.2%, 168/522) and information (20.7%, 108/522). Additionally, the percentage of social support pages varied considerably by health condition. For example, several health conditions were represented by few or no social support pages (HIV, AIDS, HPV, herpes, diarrhea, flu symptoms, pneumonia, anemia, blood pressure, and spine) and were largely represented by information and marketing/promotion pages. In contrast, five health conditions (stroke, lupus, cancer, breast cancer, stomach) were represented by 20-67% by social support pages.

Direct comparisons with other studies are difficult due to the differences in the focus on pages versus groups, classification schemes, and the range of health conditions examined. However, the relative lack of pages devoted to social support that we found is consistent with the findings of Bender et al [11] of Facebook breast cancer groups. Although we found a greater percentage of breast cancer pages devoted to social support (22.6%, 7/31) than Bender et al among groups (7%), they found that groups devoted to fundraising (45%) and raising awareness (38%) were most common. In contrast, Farmer et al [14] found that support groups made up a substantial percentage of groups for the 11 most prevalent non-communicable diseases on Facebook. They found that patient groups accounted for 47% of groups, followed by patient/caregiver support groups (28%), and fundraising groups (19%).

The relatively low percentage of social support pages for some health conditions may be due to the higher level of stigma associated with these conditions (ie, HIV, AIDS, HPV, herpes) compared to non-communicable diseases (ie, stroke, lupus, cancer, breast cancer). Rains [25] found that anonymity was one strategy used by people who are embarrassed by their illness and that people with high levels of online anonymity disclosed more health experiences. The lack of anonymity on Facebook may pose a barrier to people’s willingness to disclose information about their health condition or to provide open support to other users and limit the effectiveness of public health interventions for some health conditions. Further research is needed to explore how perceived stigma and illness-related embarrassment influences people’s willingness to disclose information and express social support.

### Engagement

Third, we found that engagement measured by Likes was disproportionate to the number of pages in each category. For example, general support and marketing/promotion pages comprised a larger percentage of Likes than would be expected given the percentage of pages. General support pages represented only 3.6% (19/522) of pages but comprised 35.89% (7,964,328/22,191,633) of Likes. Similarly, marketing/promotion represented 32.2% (168/522) of pages but comprised 46.73% (10,371,169/22,191,633) of Likes. In contrast, patient support and information/awareness pages were underrepresented in Likes compared to percentage of pages, while Wikipedia pages received no Likes.

The greater engagement with general support and marketing/promotion pages versus patient support and information/awareness pages may have to do with what Facebook users view as appropriate use and activities on the site. Lampe et al [26] studied the perception of Facebook’s value as an information source and found that on average, users did not find it appropriate to seek information on Facebook and were not likely to make extensive use of the site for information seeking. Another reason may be that marketing/promotion pages have commercial interest in gaining popularity and may employ methods like Facebook ads or viral campaigns to increase the visibility and engagement with their pages through likes. Future research should explore users’ perceived norms surrounding the use of Facebook and how these norms impact disclosure of health conditions, seeking health information, and providing social support to people who are ill.

### Limitations and Future Research

Although this study has several strengths, including the examination of the search results for 20 health conditions and nearly 1000 Facebook pages and a content categorization scheme based on previous health communication research, it has several limitations.

First, in the time since we collected our data, Facebook has modified its search function. Starting in January 2013, Facebook began to roll out a new “graph search”, which became available to all of Facebook’s English (US) users by the end of July. The new search function includes three visible changes. First, search results are formatted slightly differently: profile pictures and fonts are larger and more prominent. Second, the search results now include a column on the right side of the page, which features the name, profile picture, and cover picture for the top search result as well as Web searches for the search term. Finally, search listings now include a line for each page that indicates pages that “people also like.”

In practice, these changes appear to have little impact on searches regarding health conditions. Even so, the new search may have network effects that will impact future search results and which pages users are likely to view. In particular, the “people also like” feature may guide users to certain pages. Given that it lists similar types of pages as well (other non-profit organizations in the example above), it may also help users find certain types of pages. Future research will be required to examine how changes in search engines impact users’ ability to find relevant information and pages.

Second, we evaluated the representation of health conditions only on Facebook pages and we did not examine private messages or private groups as we were interested in what is made public to all users when searching for health conditions on Facebook. Private messaging and groups might be more appropriate channels for communicating about sensitive health topics and warrant future research. This limitation is not unique to our study; previous research on health conditions among Facebook groups has focused on public groups and messages. Additionally, our data were collected over a limited time period and for only the first 50 pages returned in the search results. A more comprehensive set of data may yield evidence of longitudinal or seasonal patterns in the representation of health conditions that we were not able to detect.

Third, we did not attempt to formally evaluate the accuracy of the informational content of pages. Thus, pages that were categorized as relevant may vary greatly in the utility of the information provided for differing health conditions. Future research on the quality of content across key health conditions may highlight critical topics of misinformation and be used to support interventions designed to correct and/or counter poor information resources.

Fourth, our descriptive analysis does not provide any data on the characteristics of Facebook members who searched for health conditions or how they used the information they found. Future research should examine how people make use of Facebook as one element of a communication ecology to address their informational needs and to garner social support, and how this usage impacts their health care utilization, self-care, and health outcomes.

### Conclusions

The rapid growth and diffusion of social media and SNS during the past 10 years has created new opportunities for people to find and share information about a wide variety of health conditions. Facebook is the most widely used SNS in the United States; however, little is known about the diversity of health conditions represented on Facebook. This research represents the first attempt to comprehensively describe the content and level of user engagement with health conditions on Facebook pages. Our findings provide useful baseline information and several insights to inform future research and interventions designed to improve public health. We found that a search of Facebook for common health conditions provided a large number of irrelevant pages. In addition, most pages were devoted to marketing/promotion and relatively few pages were devoted to social support. Social support was especially underrepresented in pages for health conditions for communicable diseases. Public health interventions using Facebook will need to be designed to ensure relevant information is easy to find and with an understanding that stigma associated with some health conditions may limit the utility of Facebook as a channel for health communication. This line of research merits further investigation as Facebook and other SNS continue to evolve over the coming years.

## Abbreviations

ADHD: attention deficit hyperactivity disorder
AIDS: acquired immunodeficiency syndrome
HIV: human immunodeficiency virus
HPV: human papillomavirus
IRR: interrater reliability
SNS: social networking sites

## References

[1] Fox S, Duggan M (2013). Pew Internet and American Life Project.

[2] Hesse BW, Moser RP, Rutten LJ (2010). Surveys of physicians and electronic health information. N Engl J Med.

[3] Cotten SR (2001). Implications of internet technology for medical sociology in the new millennium. Sociological Spectrum.

[4] Moorhead SA, Hazlett DE, Harrison L, Carroll JK, Irwin A, Hoving C (2013). A new dimension of health care: systematic review of the uses, benefits, and limitations of social media for health communication. J Med Internet Res.

[5] Brenner J (2013). Pew Internet and American Life Project.

[6] Duggan M, Brenner J Pew Internet and American Life Project.

[7] (2014). Facebook.

[8] Greene JA, Choudhry NK, Kilabuk E, Shrank WH (2011). Online social networking by patients with diabetes: a qualitative evaluation of communication with Facebook. J Gen Intern Med.

[9] Ahmed OH, Sullivan SJ, Schneiders AG, McCrory P (2010). iSupport: do social networking sites have a role to play in concussion awareness?. Disabil Rehabil.

[10] Gajaria A, Yeung E, Goodale T, Charach A (2011). Beliefs about attention-deficit/hyperactivity disorder and response to stereotypes: youth postings in Facebook groups. J Adolesc Health.

[11] Bender JL, Jimenez-Marroquin MC, Jadad AR (2011). Seeking support on Facebook: a content analysis of breast cancer groups. J Med Internet Res.

[12] Thoren EM, Metze B, Bührer C, Garten L (2013). Online support for parents of preterm infants: a qualitative and content analysis of Facebook 'preemie' groups. Arch Dis Child Fetal Neonatal Ed.

[13] De la Torre-Díez I, Díaz-Pernas FJ, Antón-Rodríguez M (2012). A content analysis of chronic diseases social groups on Facebook and Twitter. Telemed J E Health.

[14] Farmer AD, Bruckner Holt CE, Cook MJ, Hearing SD (2009). Social networking sites: a novel portal for communication. Postgrad Med J.

[15] Frankhauser D (2013). Mashable.com.

[16] Warble A (2010). Notes by Facebook Engineering.

[17] Sajadi KP, Goldman HB (2011). Social networks lack useful content for incontinence. Urology.

[18] Dobransky K, Hargittai E (2012). Inquiring minds acquiring wellness: uses of online and offline sources for health information. Health Commun.

[19] Percheski C, Hargittai E (2011). Health information-seeking in the digital age. J Am Coll Health.

[20] Robinson L (2009). A taste for the necessary: a Bourdieuian approach to digital inequality. Information, Communication & Society.

[21] Hargittai E, Hinnant A (2008). Digital inequality: differences in young adults' use of the Internet. Communication Research.

[22] DiMaggio P, Hargittai E, Celeste C, Shafer S, Neckerman KM (2004). Digital inequality: from unequal access to differentiated use. Social Inequality.

[23] Viswanath K, Thompson GE, Michell F, Williams MB (2006). Public communications and its role in reducing and eliminating health disparities. Examining the health disparities research plan of the National Institutes of Health: unfinished business.

[24] Viswanath K (2005). Science and society: the communications revolution and cancer control. Nat Rev Cancer.

[25] Rains SA (2014). The implications of stigma and anonymity for self-disclosure in health blogs. Health Commun.

[26] Lampe C, Vitak J, Gray R, Ellison N (2012). Perceptions of Facebook's value as an information source. Proceedings of the SIGCHI Conference on Human Factors in Computing Systems.

